# Gynaecological morbidity among HIV positive pregnant women in Cameroon

**DOI:** 10.1186/1742-4755-5-3

**Published:** 2008-07-03

**Authors:** Enow R Mbu, Eugene J Kongnyuy, FX Mbopi-Keou, Rebecca N Tonye, Philip N Nana, Robert JI Leke

**Affiliations:** 1 Department of Obstetrics and Gynaecology, Faculty of Medicine and Biomedical Sciences, University of Yaounde I, Cameroon; 2 Child and Reproductive Health Group, Liverpool School of Tropical Medicine, UK; 3 Department of Microbiology and Infectious Diseases, Faculty of Medicine and Biomedical Sciences, University of Yaounde I, Yaounde, Cameroon; 4 Yaounde Central Hospital Maternity, Yaounde Central Hospital, Cameroon

## Abstract

**Objective:**

To compare the prevalence of gynaecological conditions among HIV infected and non-infected pregnant women.

**Methods:**

Two thousand and eight (2008) pregnant women were screened for HIV, lower genital tract infections and lower genital tract neoplasia at booking antenatal visit.

**Results:**

About 10% (198/2008) were HIV positive. All lower genital tract infections except candidiasis were more prevalent among HIV positive compared to HIV negative women: vaginal candidiasis (36.9% vs 35.4%; *p *= 0.678), Trichomoniasis (21.2% vs 10.6%; *p *< 0.001), gonorrhoea (10.1% vs 2.5%; *p *< 0.001), bacterial vaginosis (21.2% vs 15.2%; *p *= 0.026), syphilis (35.9% vs 10.6%; *p *< 0.001), and *Chlamydia trachomatis *(38.4% vs 7.1%; *p *< 0.001). Similarly, HIV positive women more likely to have preinvasive cervical lesions: low-grade squamous intraepithelial lesion (SIL) (18.2% vs 4.4%; *p *< 0.001) and high-grade squamous intraepithelial lesion (12.1% vs 1.5%; *p *< 0.001).

**Conclusion:**

We conclude that (i) sexually transmitted infections (STIs) are common in both HIV positive and HIV negative pregnant women in Cameroon, and (ii) STIs and preinvasive cervical lesions are more prevalent in HIV-infected pregnant women compared to their non-infected compatriots. We recommend routine screening and treatment of STIs during antenatal care in Cameroon and other countries with similar social profiles.

## Introduction

There are three categories of reproductive morbidity – obstetric, gynaecological and contraceptive morbidity. Gynaecological morbidity has been defined as 'any condition, disease or dysfunction of the reproductive system that is not related to pregnancy, abortion or childbirth but may be related to sexual behaviour' [1].

Gynaecological conditions are frequent in women infected with Human Immunodeficiency Virus (HIV). Both pregnancy and HIV/AIDS predispose women to certain gynaecological conditions because of modification of the immune system [2,3]. The Centers for Disease Control and Prevention (CDC) classification system for HIV infection includes several gynaecological conditions such as persistent, frequent or poorly responsive episodes of vaginal candidiasis, moderate or severe cervical intraepithelial neoplasia (CIN) and pelvic inflammatory disease (PID), chronic herpes simplex virus ulcers and invasive cervical cancer [4].

Korn and colleagues reviewed gynaecological disease in women infected with HIV and identified five categories: (i) lower genital tract neoplasia, (ii) pelvic inflammatory disease, (iii) menstrual disorders, (iv) sexually transmitted infections, (v) vaginitis and (vi) adverse effects of contraception [5].

Three of these categories are relevant in pregnancy, namely lower genital tract neoplasia, sexually transmitted infections and vaginitis. Pelvic inflammatory disease and menstrual disorders are not conditions we encounter in pregnancy.

Cameroon is one of the sub-Saharan African countries with a high prevalence of HIV (6.8% among women of reproductive age) [6]. Only HIV and syphilis are screened routinely during antenatal care in Cameroon, despite the fact that HIV is known to be associated with other sexually transmitted infections [7]. After two decades of research on HIV, the prevalence of gynaecological conditions among HIV positive pregnant women is still unknown in Cameroon.

Gynaecological morbidity in HIV infected pregnant women has been largely ignored by research despite the frequency and severity of these conditions [8]. Previous research has focused mainly on gynaecological diseases in HIV positive non-pregnant women [9].

The aim of this study was to describe the frequency of gynaecological conditions among pregnant women and to compare the prevalence of these conditions among HIV positive and HIV negative pregnant women.

## Methods

The study was conducted in a referral hospital (Yaounde Central Hospital) in Cameroon between January 2006 and December 2006. The study was approved by the Ethics Committee of the Faculty of Medicine and Biomedical Sciences (University of Yaounde I).

All data were collected during the booking antenatal visit. Pregnant women who received antenatal care during the period of study and gave consent to participate in the study were eligible for inclusion.

A detailed gynaecological and obstetrical history was taken followed by a clinical examination during the booking visit and any clinically detectable lesions were recorded. Laboratory investigations were carried out for gynaecological diseases known to be associated with HIV: cervical intraepithelial lesions (CIN), sexually transmitted infections and vaginitis. Only syphilis and HIV are screened routinely during antenatal care in Cameroon.

Determine rapid test (Abbott Laboratories, IL, USA) was used to screen for HIV. All reactive and doubtful samples were confirmed with the Western Blot test. Vaginal swabs were taken and a wet mount prepared in normal saline immediately after collection and examined for the presence of clue cells, yeast cells and motile *Trichomonal vaginalis*. Direct microscopic examination was performed on Gram-stained genital swabs for the detection of leukocytes and Gram-negative diplococci. *Neisseria gonorrhea *was isolated by inoculation of the genital swab on modified Thayer Martin media followed by incubation in a candle extinction jar at 36°C for 24–48 hour. Identification of isolates was performed on the basis of colony morphology, visualization of Gram-negative diplococci, positive oxidase reaction and sugar fermentation tests. Bacterial vaginosis was diagnosed according to Amsel criteria [10]. Candidiasis was diagnosed by isolation of the Gram-positive yeast-like cells on Saboraud's dextrose agar and confirmed by a positive germ tube test. Syphilis was diagnosed if both Venereal Disease Research Laboratory (VDRL) and Treponema Pallidum Hemagglutination Assay (TPHA) were positive. A positive VDRL and negative TPHA was considered false positive. A positive TPHA test alone was interpreted as evidence of a past infection. *Chlamydia trachomatis *was diagnosed by Micro Trak (Syva Co., Palo Alto, Calif.) direct immunofluorescence test of cervical scrapings [11].

Data was entered into SPSS Version 13. Descriptive statistics such as proportions were analysed and presented. Univariate analyses, using odds ratio (OR) and Chi-square test for association were conducted to assess the association between HIV status and gynaecological conditions. All significance tests were two-tailed and statistical significance was defined at the 5% alpha level.

## Results

Two thousand and eight (2008) women participated in the study. Six hundred and thirty eight women (31.8%) had their booking visit during the first trimester, 1210 women (60.3%) during the second trimester and 160 women (8.0%) women during the third trimester.

### Socio-demographic characteristics of study population

Socio-demographic characteristics of the study participants are shown in Table 1. The mean age was 28 years (standard deviation [SD] = 9.1). A majority (58.0%) of the women were aged 20–29 years. About 20.0% were nulliparous women, while 38.0% had four previous deliveries or more. Most of them were married (58.0%) and had secondary education or higher (58.2%). Up to 90.0% of the study population came from urban area and the rest were from rural areas closed to the capital city (Yaounde).

**Table 1:** Socio-demographic characteristics of study population.

**Characteristic**	**HIV Positive ** **(%) ** **(N = 198)**	**HIV negative** **(%) ** ** (N = 1810)**	**Total ** **(%) ** **(N = 2008)**
**Age**			
<20 years	25/198 (12.6)	380/1810 (21.0)	405/2008 (20.2)
20–29 years	120/198 (60.6)	1044/1810 (57.7)	1164/2008 (58.0)
≥ 30 years	53/198 (26.8)	386/1810 (21.3)	439/2008 (21.9)
**Parity**			
0	30/198 (15.2)	375/1810 (20.7)	407/2008 (20.3)
1–3	78/198 (39.4)	766/1810 (42.3)	844/2008 (42.0)
≥ 4	90/198 (45.5)	667/1810 (36.9)	757/2008 (37.7)
**Marital status**			
Single	75/198 (37.9)	769/1810 (42.5)	844/2008 (42.0)
Married	123/198 (62.1)	10571810 (58.4)	1164/2008 (58.0)
**Education**			
No formal education	29/198 (14.6)	168/1810 (9.3)	197/2008 (9.8)
Primary	69/198 (34.8)	573/1810 (31.7)	642/2008 (32.0)
Secondary or higher	100/198 (50.5)	1069/1810 (59.1)	1169/2008 (58.2)
**Residence**			
Rural	8/198 (4.0)	189/1810 (10.4)	196/2008 (9.8)
Urban	190/198 (96.0)	1622/1810 (89.6)	1812/2008 (90.2)

### Prevalence of HIV

Of the 2008 women tested for HIV, 198 were HIV positive, giving an overall HIV prevalence of 9.9% among pregnant women during their booking ANC visit.

### Prevalence of gynaecological morbidity

The prevalence of gynaecological conditions among pregnant women by HIV status is presented in Table 2. All genital tract infections except candidiasis were more prevalent among HIV positive compared to HIV negative women: vaginal candidiasis (36.9% vs 35.4%), Trichomoniasis (21.2% vs 10.6%), gonorrhoea (10.1% vs 2.5%), bacterial vaginosis (21.2% vs 15.2%) and *Chlamydia trachomatis *(38.4% vs 7.1%). The prevalence of syphilis was also higher among HIV positive women (35.9% vs 10.6%). Similarly, HIV positive women had a higher prevalence of preinvasive cervical lesions: low-grade squamous intraepithelial lesion (SIL) (18.2% vs 4.4%) and high-grade squamous intraepithelial lesion (12.1% vs 1.5%).

**Table 2:** Gynaecological conditions among pregnant women at booking visit by HIV status.

**Gynaecological condition**	**HIV positive ** **(%) ** **(N = 198)**	**HIV negative ** **(%) ** **(N = 1810)**	**Prevalence rate** **ratio** ** (95% CI)**	**p-value**
Candidiasis	73 (36.9)	640 (35.4)	1.04 (0.86–1.26)	0.678
Trichomoniasis	42 (21.2)	192 (10.6)	2.00 (1.48–2.70)	<0.001
Gonorrhoea	20 (10.1)	46 (2.5)	3.98 (2.40–6.58)	<0.001
Bacterial vaginosis	42 (21.2)	274 (15.2)	1.40 (1.05–1.87)	0.026
Syphilis	71 (35.9)	192 (10.6)	3.37 (2.69–4.23)	<0.001
Chlamydia infection	76 (38.4)	128 (7.1)	5.43 (4.26–6.92)	<0.001
Low-grade SIL	36 (18.2)	80 (4.4)	4.11 (2.86–5.93)	<0.001
High-grade SIL	24 (12.1)	27 (1.5)	8.13 (4.78–13.80)	<0.001

SIL = squamous intraepithelial lesion

## Discussion

This study compared the prevalence of gynaecological morbidity among pregnant women, according to their HIV serostatus identified during antenatal care. Sexually transmitted infections (STIs) and preinvasive cervical lesions were found to be more prevalent among HIV infected pregnant women compared to their non-infected counterparts.

The high prevalence of STIs among HIV positive women has previously been reported [5]. Sexually transmitted infections producing ulcerative lesions such as syphilis, chancroid and genital herpes simplex virus (HSV) are associated with a higher rate of HIV transmission [5]. Non-ulcerative STIs are also associated with a three- to five-fold increase in the risk of HIV acquisition [7]. Chaisilwattana et al (1997) reported a higher prevalence of gonorrhoea and *Chlamydia trachomatis *among HIV pregnant women [12]. Gonorrhoea increases the risk for pelvic inflammatory disease, infertility, ectopic pregnancy, and acquisition and transmission of human immunodeficiency virus (HIV) [13]. HIV infection and STIs have common risk factors, and STIs increase the risk of acquisition of HIV. Some studies failed to find an association between HIV and STIs, and this has been explained by insufficient statistical power to detect the association [14].

It is not clear whether HIV infection increases the risk of acquisition of vaginal infections, but these conditions are common among sexually active women. *Candida albucans *and other *Candida *species are known to cause symptomatic infections in women with certain risk factors such as diabetes mellitus, broad spectrum antibiotics and immunosuppression therapy [5]. Vaginal candidiasis is also frequent in pregnancy. Leroy et al (1995) reported a similar prevalence of vaginal candidiasis among HIV-infected pregnant women (22.3%) and non-infected pregnant women (20.1%) [15]. The prevalence of vaginal candidiasis in HIV-infected women depends on the CD4 count. Burns et al (1997) reported a 3-fold increase in vaginal candidiasis among HIV-infected women with low CD4 counts compared to HIV-infected women with normal CD4 count during pregnancy [16]. Apart from Candida spp, we reported an increase in the prevalence of bacterial vaginosis (BV) among HIV pregnant women. Bacterial vaginosis increases susceptibility to infection by HIV and other genital tract pathogens, but it is not clear whether HIV increases the risk of developing BV [17].

We reported an 8-fold increase in high grade SIL among HIV infected pregnant women compared to their non-infected compatriots. A 5-fold increase in cervical intraepithelial neoplasia among HIV-infected women has been described previously [5]. There is evidence that the severity of preinvasive cervical lesion is related to the degree of immunosuppression, suggesting that immunosupprsession contributes (at least in part) to the risk of developing preinvasive lesion [18,19]. Some studies suggest that the high prevalence of preinvasive cervical lesion may be related to concurrent risk factors related to the mode of transmission of HIV [20]. Ahr et al (2006) reported that HIV positive women with low CD4 count had a higher prevalence of human papilloma virus (HPV) and preinvasive cervical lesion was more frequent in women with HPV [21]. HPV is a known causative agent for preinvasive and invasive lesions of the cervix.

This study has some limitations. First of all, we relied on clinically detectable lesions for the diagnosis of genital herpes simplex virus and chancroid. Secondly, high levels of cardiolipin antibodies associated with HIV infection could interfere with serologic tests for syphilis and give false positive results [5].

We conclude that (i) STIs are common in both HIV positive and HIV negative women in Cameroon, and (ii) STIs and preinvasive cervical lesions are more prevalent in HIV-infected pregnant women compared to their non-infected compatriots. We recommend routine screening and treatment of STIs during antenatal care in Cameroon and countries with similar social profiles.

## Competing interests

The authors declare that they have no competing interests.

## Authors' contributions

ERM, EJK conception, design, drafting of the protocol, analysis and interpretation. Both authors contributed equally to the realisation of this paper, FXMK, RNT, PNN, RJLL critically revising the final article for important intellectual content. All authors read and approved the manuscript.
